# Efficient Mutagenesis by Cas9 Protein-Mediated Oligonucleotide Insertion and Large-Scale Assessment of Single-Guide RNAs

**DOI:** 10.1371/journal.pone.0098186

**Published:** 2014-05-29

**Authors:** James A. Gagnon, Eivind Valen, Summer B. Thyme, Peng Huang, Laila Ahkmetova, Andrea Pauli, Tessa G. Montague, Steven Zimmerman, Constance Richter, Alexander F. Schier

**Affiliations:** 1 Department of Molecular and Cellular Biology, Harvard University, Cambridge, Massachusetts, United States of America; 2 Department of Biochemistry and Molecular Biology, University of Calgary, Calgary, Alberta, Canada; 3 Center for Brain Science, Harvard University, Cambridge, Massachusetts, United States of America; 4 Broad Institute of Massachusetts Institute of Technology and Harvard, Cambridge, Massachusetts, United States of America; 5 Harvard Stem Cell Institute, Harvard University, Cambridge, Massachusetts, United States of America; Texas A&M University, United States of America

## Abstract

The CRISPR/Cas9 system has been implemented in a variety of model organisms to mediate site-directed mutagenesis. A wide range of mutation rates has been reported, but at a limited number of genomic target sites. To uncover the rules that govern effective Cas9-mediated mutagenesis in zebrafish, we targeted over a hundred genomic loci for mutagenesis using a streamlined and cloning-free method. We generated mutations in 85% of target genes with mutation rates varying across several orders of magnitude, and identified sequence composition rules that influence mutagenesis. We increased rates of mutagenesis by implementing several novel approaches. The activities of poor or unsuccessful single-guide RNAs (sgRNAs) initiating with a 5′ adenine were improved by rescuing 5′ end homogeneity of the sgRNA. In some cases, direct injection of Cas9 protein/sgRNA complex further increased mutagenic activity. We also observed that low diversity of mutant alleles led to repeated failure to obtain frame-shift mutations. This limitation was overcome by knock-in of a stop codon cassette that ensured coding frame truncation. Our improved methods and detailed protocols make Cas9-mediated mutagenesis an attractive approach for labs of all sizes.

## Introduction

There is an urgent need for precise, predictable, inexpensive, and easy-to-use genome engineering tools that are applicable to a wide range of model organisms and cell types. Existing genome editing tools such as zinc-finger nucleases (ZFNs) and transcription activator-like effector nucleases (TALENs) have enabled reverse genetics in many systems [1], but their widespread adoption has been limited by the cost of commercially available reagents, a requirement for substantial molecular cloning, and/or unpredictable activity. The CRISPR/Cas system of bacterial adaptive immunity has been recently applied to genome editing in many model organisms [2], [3]. Briefly, the *S. pyogenes* Cas9 enzyme uses a short CRISPR RNA that directs cleavage through its complementarity to 20 bases of genomic DNA sequence, and a trans-activating RNA that induces sequence-specific double-strand breaks in targeted DNA adjacent to an NGG trinucleotide known as the protospacer adjacent motif (PAM). The CRISPR RNA and the trans-activating RNA can be fused to generate a single-guide RNA (sgRNA) sufficient for site-directed cleavage of target DNA [4]. This simplified system has been implemented in a variety of *in vivo* settings, resulting in efficient mutagenesis by NHEJ-mediated small insertions or deletions [5]–[9]. A primary advantage of the Cas9 method over existing methods is that mutagenesis can be directed to diverse genomic locations by simply exchanging the sgRNA, without the need to reengineer the Cas9 enzyme. This flexibility and the published rates of mutagenesis (comparable or superior to ZFNs and TALENs) make Cas9 an attractive system for site-directed mutagenesis.

Despite the rapid progress in Cas9-mediated genome engineering, several questions and limitations remain. Only a small number of loci have been targeted in model organisms. For example, in zebrafish [9]–[15], only 21 genes have been targeted. Thus, the sequence rules for sgRNA effectiveness and the spectrum of generated alleles remain unknown. Moreover, the optimal methods for generating and delivering sgRNAs and Cas9 enzyme are still unclear. For example, in zebrafish, the current design of sgRNAs restricts the targeting range to a subset of the genome and it has been claimed that Cas9 protein injection generates only modest indel frequencies [9], [11]. Here we address these questions by targeting a large number of genomic loci in zebrafish using an optimized Cas9 system and detecting mutations with next-generation sequencing.

## Materials and Methods

Detailed protocols for implementation are available as Supplemental Protocols S1.

### Ethics Statement

All vertebrate animal work was performed at the facilities of Harvard University, Faculty of Arts & Sciences (HU/FAS). The HU/FAS animal care and use program maintains full AAALAC accreditation, is assured with OLAW (A3593-01), and is currently registered with the USDA. This study was approved by the Harvard University/Faculty of Arts & Sciences Standing Committee on the Use of Animals in Research & Teaching under Protocol No. 25–08.

### Target site selection for the initial screen

Briefly, target sites were selected in exons across the genome that matched the sequence GG-N_19_-GG, GA-N_19_-GG, or AG-N_19_-GG. These sites were checked for uniqueness in RefSeq protein coding regions using Bowtie [16] and the initially defined specificity rules [6], which suggested an intolerance for mismatches in the 3′ twelve bases of the target site.

### Cas9 cloning and protein expression

For *in vivo* expression of Cas9, the Cas9 open reading frame was amplified from hCas9 [5] and cloned into the pCS2 vector to generate pCS2-Cas9. For *in vitro* expression of Cas9, the hCas9 open reading frame cloned into pET-28b to generate pET-28b-Cas9-His. Cas9 protein was expressed in *E. coli* Rosetta cells (Novagen) using the auto-induction method [17], growing for 12 hours at 37°C, followed by 24 hour expression at 18°C. Purification was performed using his-tag resin (G-Biosciences). Buffers were 20 mM Tris pH 8, 30 mM Imidazole, 500 mM NaCl for washes and 20 mM Tris pH 8, 500 mM Imidazole, 500 mM NaCl for elutions. Fractions were dialyzed into 20 mM Tris, 200 mM KCl, 10 mM MgCl_2_ and single-use aliquots were frozen in liquid nitrogen and stored at −80°C. Plasmids are available from Addgene at http://www.addgene.org/Alex_Schier/.

### sgRNA template generation and transcription

To generate templates for sgRNA transcription, gene-specific oligonucleotides containing the T7 (5′-TAATACGACTCACTATA-3′) or SP6 (5′- ATTTAGGTGACACTATA-3′) promoter sequence, the 20 base target site without the PAM, and a complementary region were annealed to a constant oligonucleotide encoding the reverse-complement of the tracrRNA tail (Supplementary Figure S1 and Table S1). The ssDNA overhangs were filled in with T4 DNA polymerase (NEB), and the resulting sgRNA template were purified using Qiaquick columns (Qiagen). sgRNAs were transcribed using Megascript kits (Ambion). All sgRNAs were then DNase treated and precipitated with ammonium actetate/ethanol. Cas9 mRNA was transcribed from linearized template DNA using mMachine SP6 kit (Ambion), DNase treated, and precipitated with lithium chloride. RNA concentration was quantified using Nanodrop spectrophotometer and diluted and aliquoted as a 500 ng/ul 20× stock (sgRNAs) or 600 ng/ul 2× stock (Cas9 mRNA).

### Fish husbandry and microinjection

For the initial screen, zebrafish TLAB strain zygotes were collected and injected through the chorion with a mix of 25 pg sgRNA, 300 pg Cas9 mRNA, and phenol red dye in a single mix. Embryos were grown to 24–30hpf and genomic DNA extracted from pools of 8–10 embryos (unless otherwise indicated) using the HotSHOT method [18]. For comparison between Cas9 mRNA and protein, higher levels of sgRNA were co-injected (200–300 pg). Cas9/sgRNA complex was formed by incubating protein with sgRNA at room temperature for 5 minutes before injection.

### Determination of somatic mutagenesis rates

To determine indel percentage using sequencing, a fusion PCR method was used to attach Illumina P5 sequencing adapters and barcodes to amplicons designed to surround the target site (Table S1). Amplicons were quantified by visualization on agarose gel before being pooled at roughly equal molar ratios. Pools of amplicons were gel extracted and sequenced with MiSeq Personal Sequencer (Illumina), 150 bp paired-end sequencing.

Each pair of reads was assigned to the correct loci based on comparing the start of the sequenced reads (corresponding to the amplicon primers) to the loci sequence. A match was required for both reads for the pair to be associated with a locus. Next, both reads were aligned to the locus using the Needleman-Wunsch algorithm. A gap open penalty of 50 and an extend penalty of 0 was used, reflecting the a priori assumption that there is either zero or one gap of unknown length present.

A paired end read was considered to originate from a cut site if it differed in length (i.e. contained an indel) by more than 1 nt in comparison to the genome reference locus sequence [19]. In the few cases where the two reads disagreed on the length, the most conservative estimate was considered. Even some wild-type loci have putative indels, likely due to genomic heterogeneity, technical artifacts or sequencing errors, and therefore overall indel frequency was calculated as the indel frequency of the reads from the injected embryos minus the expected indel frequency represented by the uninjected embryos.

Position specific biases were calculated by taking the mean cut rate for all sgRNAs that have a given nucleotide at a given position. The cut rate fold change was further calculated by taking log2 of this value over the mean cut rate for all sgRNAs.

### Stop codon cassette oligonucleotide design and injection

Each oligonucleotide contained two 20 base homology arms which flank the predicted Cas9-mediated breakpoint. These homology arms surround the stop codon cassette, with sequence 5′-GTCATGGCTAATTAATTAAGCTGTTGTAG-3′. Embryos were injected as previously described, except with a mix of Cas9 protein/sgRNA complex and 1 µM oligonucleotide.

### Verifying germline transmission of stop cassette insertion

Clutches of 20 embryos were collected from crossing adult injected fish with uninjected wild-type fish. The isolated genomic DNA was used for PCR amplification with a gene-specific primer and a primer specific to the inserted sequence.

## Results

Previous studies of ∼20 genes have shown that Cas9-mediated mutagenesis is effective in zebrafish [9]–[15]. To understand the rules underlying optimal sgRNAs, we extended these studies by targeting 122 loci in the zebrafish genome and used MiSeq deep sequencing to analyze indel frequencies and compositions. We used the previously described 5′GG-N_18_-NGG sgRNA architecture, while also relaxing the first two bases to allow 5′AG-N_18_-NGG or 5′GA-N_18_-NGG target sites for some loci, since these were previously suggested to be acceptable as initiating bases for T7 RNA polymerase [9]. We chose target sites most likely to generate a null allele in the target gene via NHEJ-generated indels. As a simple approach to reduce Cas9-mediated off-target mutations, we predicted possible off-targets in the genome using the previously identified *in vitro* and *in vivo* determined rules for targeting specificity (see Materials and Methods).

We developed a simple, scalable and cloning-independent method for generating sgRNAs that requires only a single oligonucleotide per target sequence (Figure S1). This method generates a template for *in vitro* transcription of sgRNAs containing the constant region shown to mediate the highest rates of mutagenesis [20]. Cas9 mRNA and sgRNA were co-injected into zebrafish zygotes, survival was scored at 24–30 hours post-fertilization (hpf) and genomic DNA was prepared from injected and uninjected embryos. PCR was used to amplify ∼120–300 base pairs of genomic sequence surrounding the targeted locus and to attach barcoded sequencing adapters. These amplicons were purified, pooled, and subjected to sequencing using MiSeq to obtain 1–2 million 2×150 base paired-end reads. We adapted a previously implemented algorithm to determine indel frequency [19], and improved it to account for technical artifacts and sequencing errors (see Materials and Methods).

**Figure 1:**
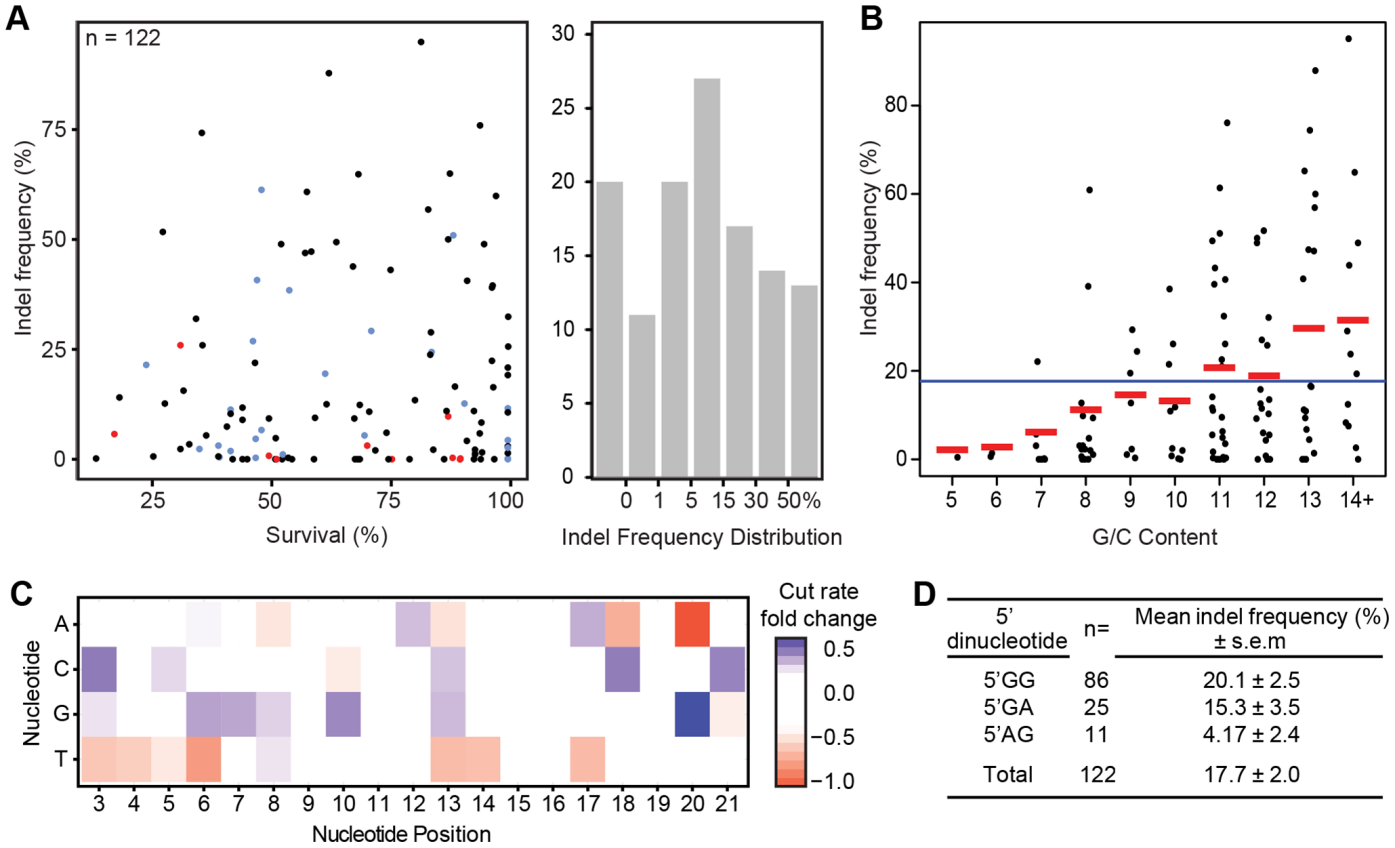
Cas9 directs a wide range of indel frequencies depending on sgRNA base composition. **A.** (left) Embryo survival at 24–30 hpf compared to indel frequency. 5′GG is black, 5′GA is blue, 5′AG is red. (right) Distribution of sgRNAs by indel frequency. **B.** G/C content of sgRNAs compared to indel frequency. Means for each category are indicated in red and the mean across all sgRNAs is indicated in blue. **C.** Heatmap plot showing position-specific effects on indel rates for nucleotide positions 3–21 (21 is the first base of the PAM). Color scale represents the increase in indel frequency of a given sgRNA containing the indicated nucleotide at the specified position. **D.** sgRNA 5′ dinucleotide pair compared to indel frequency.

85% of the sgRNAs induced somatic mutations with a mean indel frequency of 17.7%, ranging across several orders of magnitude (Figure 1A). There was no correlation between survival rate and mutagenic activity. Many sgRNAs induced indel frequencies >50%, suggestive of extensive biallelic conversion (Figure 1A). This high level of mutagenesis indicates that Cas9 is an effective method for generating targeted mutations in zebrafish, in agreement with previously published results [9], [10], [12], [13], [15]. We used our large dataset to investigate several aspects of sgRNA sequence composition to determine rules which governed effectiveness. We observed a positive correlation between G/C content and indel frequency (Figure 1B). Segregating the indel frequencies of sgRNAs by nucleotide position revealed that sgRNAs with a guanine adjacent to the PAM motifs exhibited significantly higher indel frequencies than other bases (Figure 1C). These biases confirm and extend a genome-scale assessment of Cas9 affinity for sgRNAs in tissue culture cells, which indirectly indicated similar G/C content and purine biases associated with sgRNA activity [21]. These results indicate that sgRNAs with over 50% G/C content and with a G adjacent to the PAM motif are optimal for ensuring high rates of mutagenesis.

**Figure 2:**
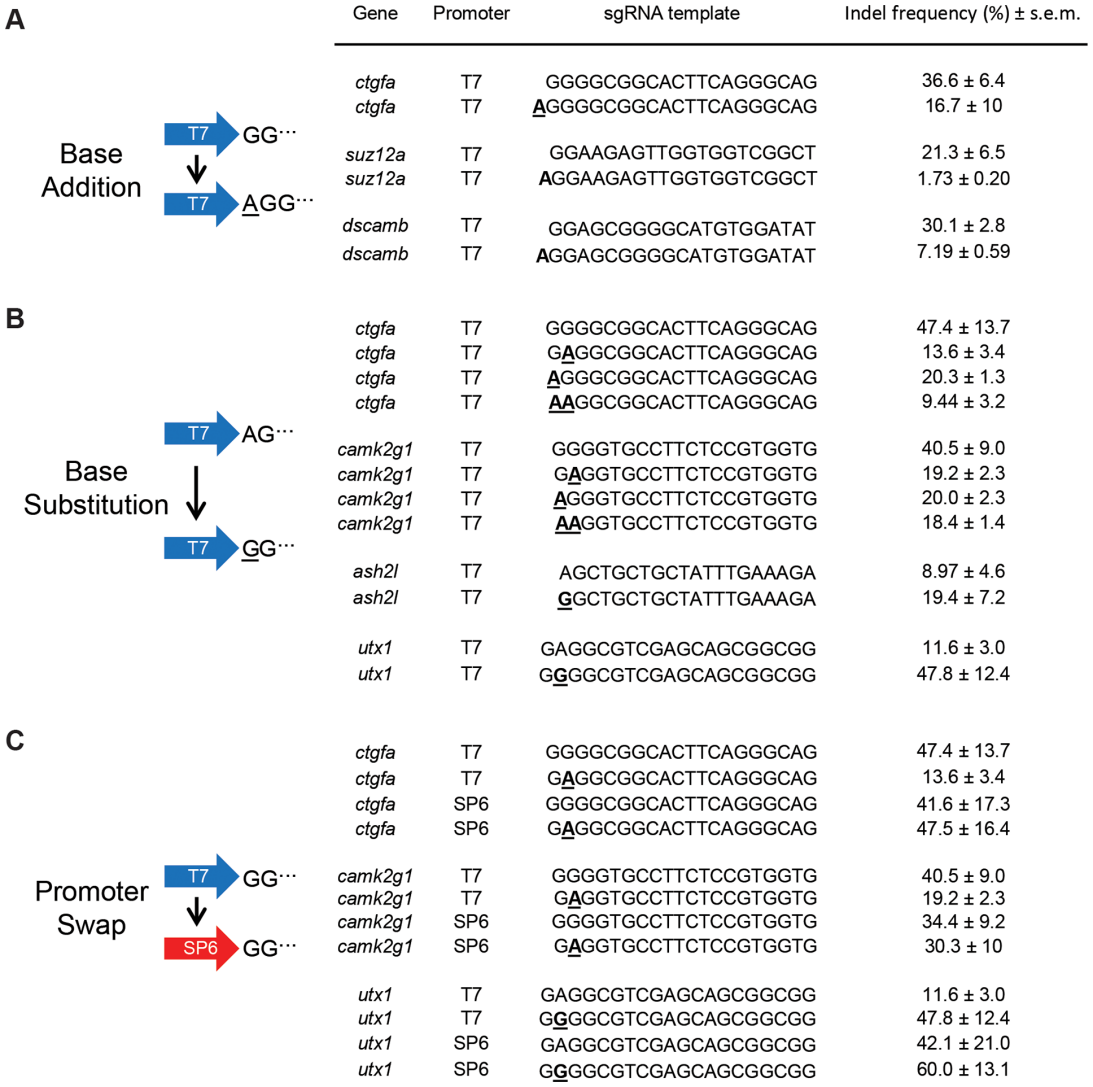
5′GG dinucleotide pair and promoter choice influence sgRNA activity. **A.** Indel frequencies of 5′GG sgRNAs modified with the addition of a 5′adenine. **B.** Indel frequencies of sgRNAs modified by substituting the 5′ dinucleotide as indicated. **C.** Indel frequencies of sgRNAs transcribed using either T7 (repeated data from above) or SP6 polymerase. Altered bases to the template are indicated in bold, mismatched bases to the genome are underlined.

We observed a bias for sgRNAs generated from transcription reactions initiating with the dinucleotide 5′ AG to have poor or no activity. While embryos injected with sgRNAs templated to initiate with a 5′AG, 5′GA, and 5′GG had similar survival rates (Figure 1A) and transcription reaction efficiencies, 5′AG sgRNAs exhibited poor mutagenic activity *in vivo*, on average 5-fold worse than 5′GG sgRNAs (Figure 1D). 5′GA sgRNAs showed a less pronounced decrease in indel frequency. To determine whether the 5′adenine directly impacts sgRNA quality, several highly active 5′GG sgRNAs were modified by adding a single adenine base to the 5′ ends, and their mutagenic capacities were assayed *in vivo*. The mutagenic activity of all three sgRNAs tested was reduced 2 to 12-fold by addition of a 5′ adenine (Figure 2A), indicating that the 5′A prevented mutagenic activity independent of genomic location. To test whether G-to-A substitutions (instead of additions) of 5′GG sgRNAs would alter activity, 5′GG sgRNAs were converted to 5′GA, 5′AG, and 5′AA. In all cases, the mutagenic activity of the sgRNA was reduced by these base substitutions (Figure 2B). Based on these results, we hypothesized that 5′GG may be sufficient to improve the mutagenic activity of poor 5′AG or 5′GA sgRNAs. We converted several ineffective 5′AG or 5′GA sgRNAs to 5′GG sgRNAs and observed up to 10-fold increases in indel frequency (Figure 2B). We conclude that (i) Cas9 can tolerate single base mismatches between the genome and the 5′end of the sgRNA, in agreement with previous studies [6], [13], and (ii) that these changes can improve activity.

We hypothesize that the poor activity of 5′AG or 5′GA sgRNAs is caused by T7-derived *in vitro* transcription errors or heterogeneity caused by attempted initiation with bases other than 5′GG, as previously described [22]–[24]. Alternatively, Cas9 enzyme may bias against 5′AG or 5′GA sgRNAs, though this seems unlikely given published activities of sgRNAs containing alternative 5′ dinucleotides generated by *in vitro* transcription-independent methods [5], [6]. To distinguish between these two possibilities, we generated several 5′GG or 5′ GA sgRNAs using SP6 RNA polymerase (Figure 2C). In contrast to T7 polymerase, the SP6 polymerase consensus initiation site is 5′GA [25]. These sgRNAs should be identical in sequence to those previously generated by T7 polymerase. When we assayed the sgRNAs for *in vivo* activity, we observed that all 5′GA sgRNAs that previously exhibited poor activity when transcribed by T7, now elicited strong mutagenesis (Figure 2C). We conclude that the previous poor activity of 5′AG or 5′GA sgRNAs was at least partially due to 5′ end transcript heterogeneity from *in vitro* transcription using T7 polymerase. Additionally, SP6-derived 5′GG sgRNAs were equally effective as T7-derived sgRNAs. Although these data come from a limited set of sgRNAs, they suggest that SP6 is a more flexible and thus superior polymerase for *in vitro* synthesis of sgRNAs.

**Figure 3:**
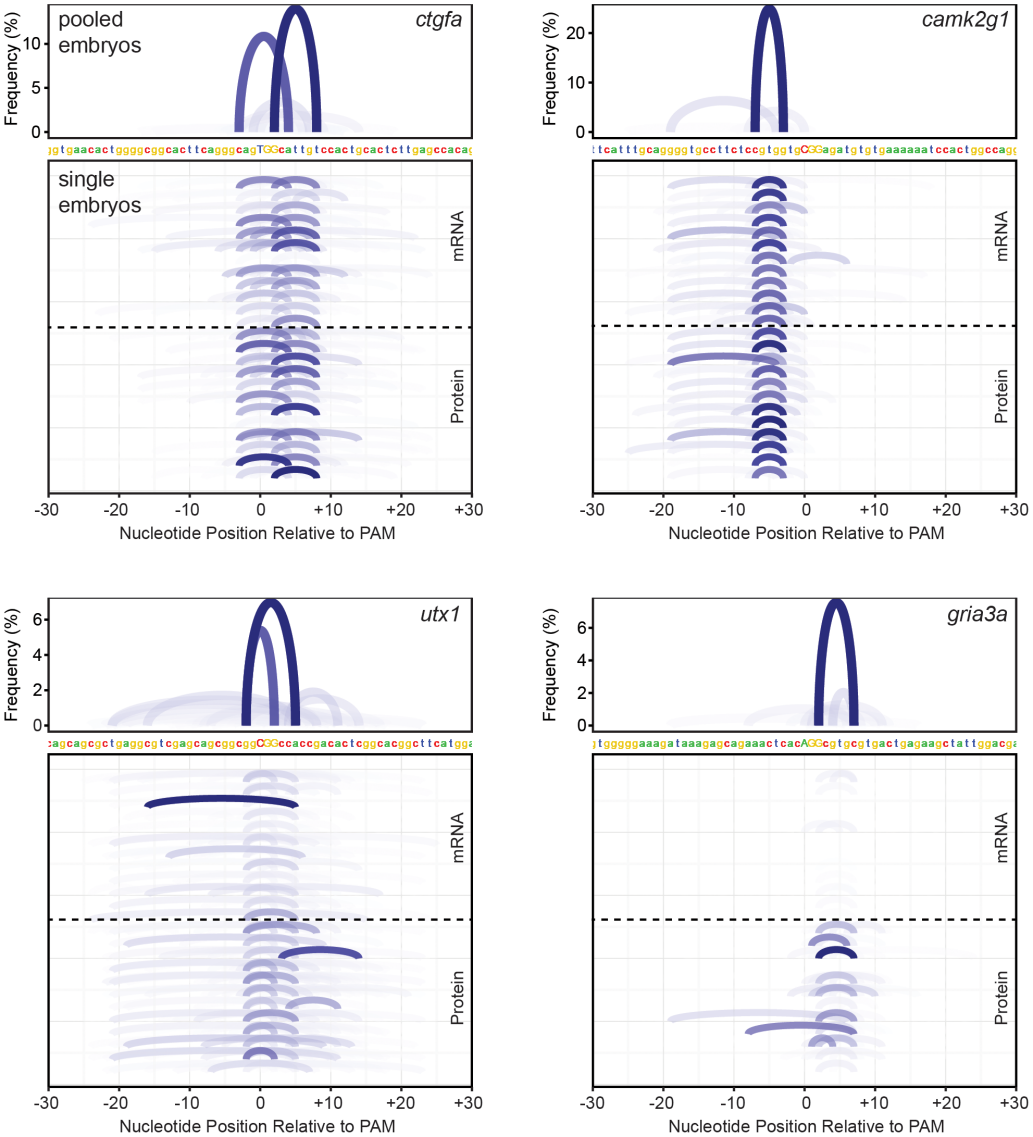
Cas9 generates minimal allele diversity. Target sites and alleles are shown for four genes for pools of embryos or single embryos (n = 12 per target with Cas9 mRNA, n = 12 per target with Cas9 protein). For each target site, the top panel is from a pool of embryos while the bottom panel represents alleles from single embryos. Each plot indicates the observed mutant alleles using arches that connect the bases surrounding the deletion. In the top panel, the y-axis and color of the arch indicate allele abundance; for ease of visualization, the bottom panel indicates allele abundance only with color. PAM is indicated on the DNA sequence in uppercase. g*ria3a* sgRNA induces only low indel frequencies when injected with Cas9 mRNA.

In addition to determining indel frequencies for targets, we used our deep sequencing data to investigate the diversity of mutant alleles. We discovered that many targets had a surprisingly small number of predominant mutant alleles, even within a pool of ten embryos (Figure 3, top panels). To exclude the possibility that PCR amplification reduced diversity during library preparation, we prepared genomic DNA from individual embryos at 24–30 hpf and profiled somatic allele diversity. For all four targets tested, specific mutant alleles were found to predominate in nearly all embryos (Figure 3, lower panels). Indeed, analysis of the initial screen sequencing data revealed that fewer than five alleles predominated for most genes, suggesting widespread allele bias after Cas9-mediated double strand breaks. We found that the recurrent somatic alleles remained predominant in the germline for the five genes that were tested. For certain genes, the predominant alleles were not generating frame-shifting mutant alleles (for example *camk2g1* in Figure 3), which makes generating mutants for protein-coding genes less predictable. These results suggest that the DNA repair machinery corrects breaks in a stereotyped and genome position-specific fashion, reducing allele diversity.

**Figure 4:**
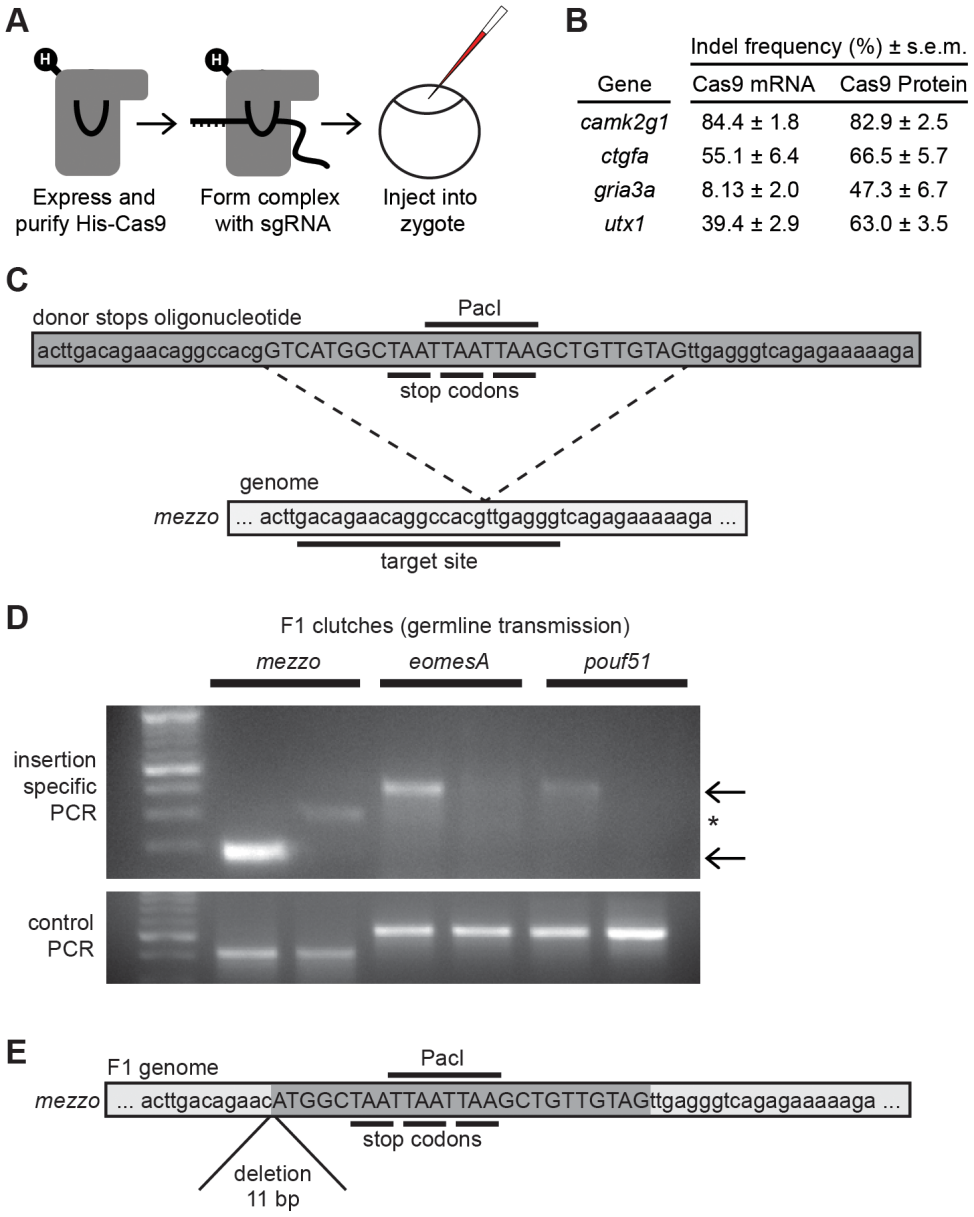
Overcoming allele biases by inserting stop codon oligonucleotide with injected Cas9/sgRNA complexes. **A.** Diagram of Cas9/sgRNA complex injection. **B.** Purified His-tagged Cas9 protein complexed with sgRNA (n = 12 embryos per target) or Cas9 mRNA and sgRNA (n = 12 embryos per target) were injected and indel frequency measured in single embryos at 1 dpf. **C.** Diagram of a stop codon oligonucleotide and genomic target site for *mezzo*. **D.** Clutches of embryos from adult injected fish were screened by PCR for germline transmission using PCR with a gene-specific primer and a primer specific to the inserted sequence (top panel), or two primers specific to the genome (bottom panel) as a positive control. Specific products indicated with arrows, non-specific product indicated with an asterix. **E.** DNA sequence of a *mezzo* insertion allele transmitted through the germline, 6/16 embryos in this clutch contained this mutation.

We developed two improvements to our Cas9 methodology. First, we hypothesized that injecting Cas9 protein would be more effective than Cas9 mRNA because it could act immediately following injection without a translational delay. However, a recent publication showed only weak mutagenesis (≤12% indel frequency) at two genes by direct injection of Cas9 protein/sgRNA complexes [11]. We expressed a His-tagged Cas9 protein in *E. coli* and purified it using standard nickel-based affinity purification (Figure 4A). We co-incubated Cas9 protein and sgRNA at room temperature to form the Cas9 protein/sgRNA complex *in vitro*, microinjected the complex into zygotes and assayed indel frequency as before. Injection of Cas9 protein/sgRNA complex was remarkably non-toxic (∼90% survival) andinduced as high or higher rates of mutagenesis *in vivo* when compared to Cas9 mRNA injection for all four targeted genes (Figure 4B). For example, *gria3a* sgRNA, which generates only moderate indel frequencies when injected with Cas9 mRNA, exhibited 6-fold better mutagenesis with complexed and injected with Cas9 protein. This suggests that the limiting step for some poor sgRNAs may be binding to Cas9 and/or *in vivo* instability. Second, we developed a method for ensuring production of a translation-terminating mutation regardless of target site DNA repair biases. Several groups have reported knock-in of small sequences, such as protein tags or loxP sites, using single-stranded DNA oligonucleotides in zebrafish [10], [13], [15]. We used a similar insertional strategy with an oligonucleotide containing stop codons in all frames and 20 nucleotide homology arms on both ends to mediate insertion (Figure 4C). Regardless of indel, frame, or orientation, upon insertion the cassette will generate an in-frame stop codon to terminate translation of the coding region. We implemented this strategy for three genes, and determined germline transmission in adults by screening pools of embryos by PCR using a gene-specific primer and an insert-specific primer. For all three genes, we observed germline transmission of the inserted stop codon cassette (Figure 4D). To confirm that the stop codon cassette generated an in-frame stop codon, we sequenced germline transmitted mutant alleles and verified that they contain the predicted insertion and a premature termination codon (Figure 4E). In conclusion, use of Cas9 protein and stop cassette oligonucleotide insertion led to consistently high rates of putative null alleles inherited through the germline.

## Discussion

Our study provides a large-scale assessment of sgRNA activity and allele diversity and introduces several improvements to the zebrafish Cas9 mutagenesis strategy. The detailed protocols for target site selection, sgRNA production, stop codon cassette design, Cas9 protein purification, injection and downstream analysis are provided on our website and in the supplement (Figure S2, Supplemental Protocols S1) and have already been successfully implemented by several other laboratories. While we tested these methods exclusively in zebrafish, they will also be adaptable to Cas9-mediated mutagenesis methods in other model organisms.

Our study provides seven advances in our understanding and implementation of Cas9-mediated mutagenesis. First, our screen of more than 100 sgRNAs revealed that sgRNAs can differ in activity across orders of magnitude. Second, our large dataset identified rules for optimal target site selection: a threshold of 50% G/C content and a guanine adjacent to the PAM. Third, we expanded the Cas9 targeting range using base substitution, confirming and extending findings from other reports [13]. Fourth, we uncovered issues with the use of T7 polymerase for *in vitro* transcription of sgRNAs, and we suggest SP6 polymerase as a superior alternative. Fifth, we demonstrated high mutation rates by directly injecting Cas9 protein/sgRNA complexes. The discrepancy between our observations and a previous report demonstrating only modest activity by Cas9 protein in zebrafish embryos [11] could be due to locus-specific effects, protein concentration differences, and/or other technical issues. Sixth, our deep sequencing data showed that certain alleles dominate after DNA repair of Cas9-mediated double strand breaks, and these predominant alleles are transmitted through the germline. Finally, knock-in mutagenesis of a stop codon cassette ensures open reading frame truncation regardless of predominant indel alleles. Use of an insertion-based mutagenesis approach for open reading frame truncations has an additional advantage: while small indel-based mutations require laborious allele-specific genotyping assays, the stop codon cassette can be used as a site for primer binding, allowing rapid genotyping.

## Supporting Information

Figure S1 **Generating sgRNAs through template assembly and** ***in vitro*** **transcription.** A gene-specific oligo is annealed to a constant oligonucleotide and filled in with DNA polymerase. This template is purified and used in an *in vitro* transcription reaction.
(TIF)

Figure S2 **Pipeline for making mutants with Cas9.** Flowchart of Cas9/sgRNA-mediated mutagenesis.
(TIF)

Table S1 **Screen results and primer design.** Genomic coordinates, target site, 5′ dinucleotide, percent survival at 24 hpf, indel frequency and primer design for amplicon sequencing for all targets in the initial screen are provided.
(XLSX)

Supplemental Protocols S1 Detailed protocols are provided in the Supplemental Protocols to guide users through each step of Cas9/sgRNA-mediated mutagenesis.
(DOCX)
