# Correction: Efficient Mutagenesis by Cas9 Protein-Mediated Oligonucleotide Insertion and Large-Scale Assessment of Single-Guide RNAs

**DOI:** 10.1371/journal.pone.0106396

**Published:** 2014-08-18

**Authors:** The PLOS ONE Staff

The fifth author’s name is misspelled. The correct name is: Laila Akhmetova. The correct citation is: Gagnon JA, Valen E, Thyme SB, Huang P, Akhmetova L, et al. (2014) Efficient Mutagenesis by Cas9 Protein-Mediated Oligonucleotide Insertion and Large-Scale Assessment of Single-Guide RNAs. PLoS ONE 9(5): e98186. doi:10.1371/journal.pone.0098186
