# Supplementary figures and images for: Efficient Mutagenesis by Cas9 Protein-Mediated Oligonucleotide Insertion and Large-Scale Assessment of Single-Guide RNAs
Source: PLoS One. 2014 May 29;9(5):e98186. doi: 10.1371/journal.pone.0098186 (PMC4038517; doi:10.1371/journal.pone.0098186)

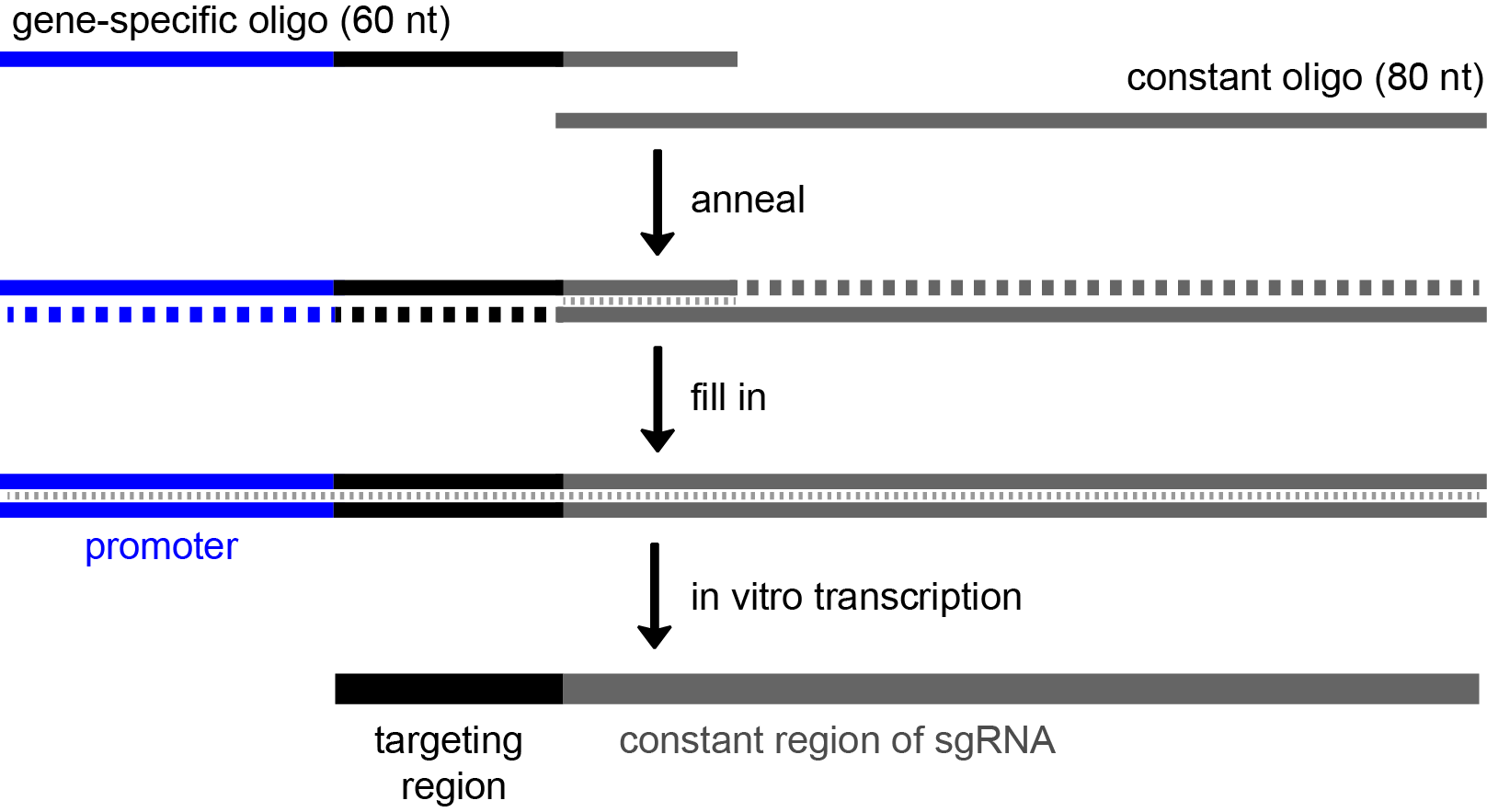

Supplement: Figure S1 — Generating sgRNAs through template assembly and in vitro transcription. A gene-specific oligo is annealed to a constant oligonucleotide and filled in with DNA polymerase. This template is purified and used in an in vitro transcription reaction. (TIF) [file pone.0098186.s001.tif]

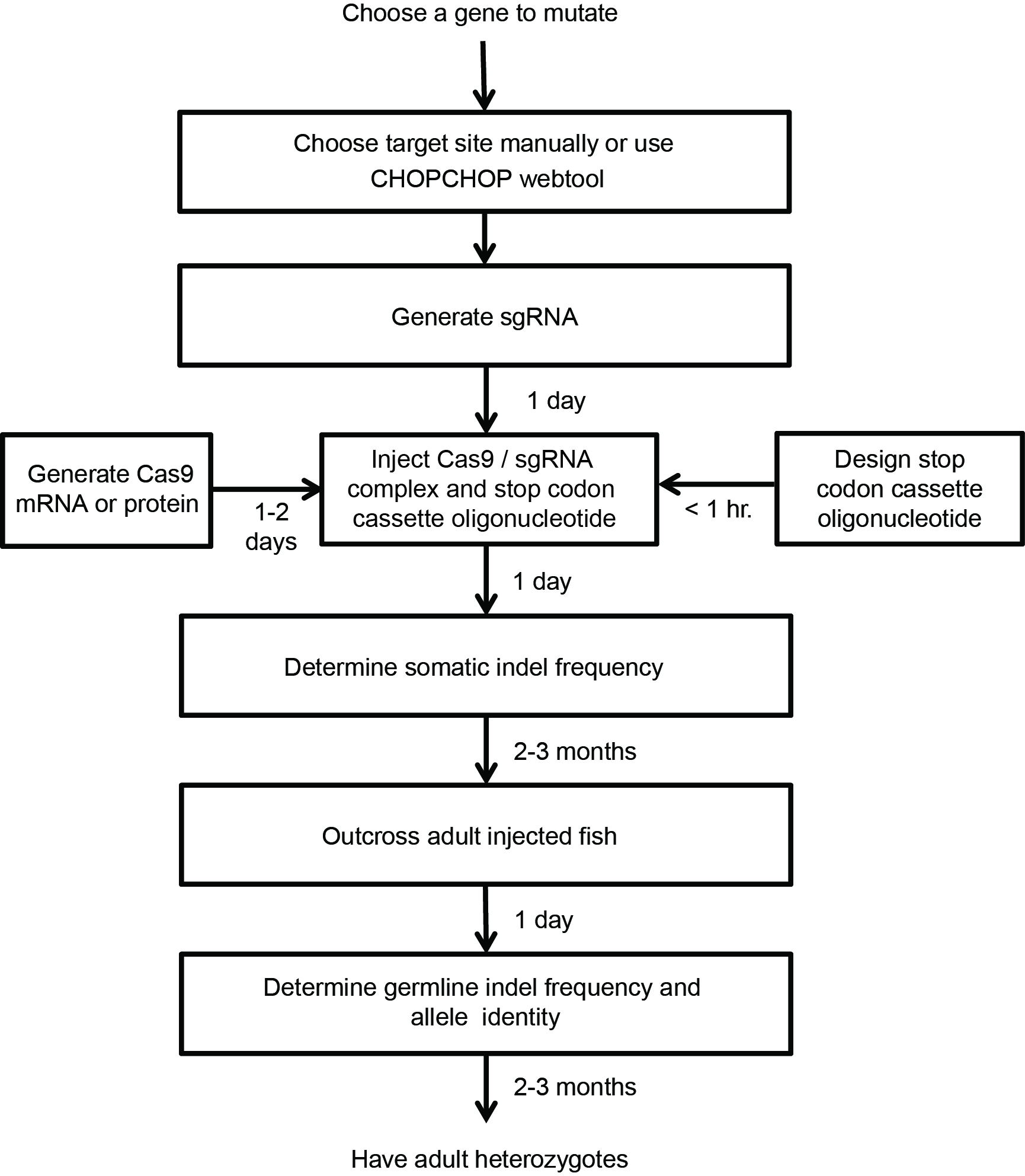

Supplement: Figure S2 — Pipeline for making mutants with Cas9. Flowchart of Cas9/sgRNA-mediated mutagenesis. (TIF) [file pone.0098186.s002.tif]
