# Supplementary material for: Efficient Mutagenesis by Cas9 Protein-Mediated Oligonucleotide Insertion and Large-Scale Assessment of Single-Guide RNAs
Source: PLoS One. 2014 May 29;9(5):e98186. doi: 10.1371/journal.pone.0098186 (PMC4038517; doi:10.1371/journal.pone.0098186)
Supplement: Supplemental Protocols S1 — Detailed protocols are provided in the Supplemental Protocols to guide users through each step of Cas9/sgRNA-mediated mutagenesis. (DOCX) [file pone.0098186.s004.docx]

Last updated April 15th 2014

There may be a more up-to-date version of this protocol on our website – <http://labs.mcb.harvard.edu/schier/index.html>

| Protocol | Page |
| --- | --- |
| Flowchart for Cas9-mediated mutagenesis | 1 |
| Target site selection | 2 |
| Generate sgRNA | 3 |
| Generate Cas9 mRNA or protein | 6 |
| Design stop codon cassette oligonucleotide | 9 |
| Inject Cas9, sgRNA, and stops oligonucleotide | 10 |
| Determine somatic indel frequency | 11 |
| Outcross adµlt injected fish | 16 |
| Determine germline indel frequency and allele identity | 16 |
| Identify adµlt heterozygous individuals | 17 |


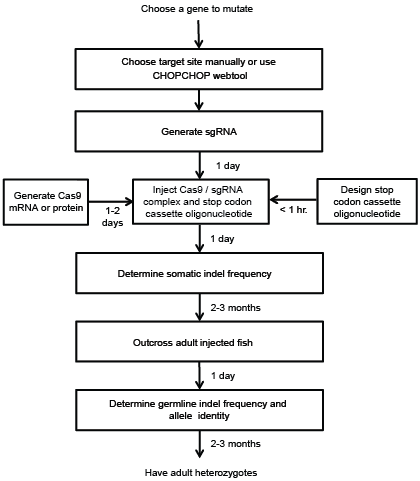


**Target site selection**

To knock out the function of a protein-coding gene, the stop codon cassette should be introduced downstream of the start codon. Transcripts resulting from a modified gene will often be degraded by nonsense-mediated decay, assuming there is a downstream exon-exon junction. Targets can be identified manually or using an online webtool such as CHOPCHOP (recommended, see below). For manual targeting, the user can search the sequence of the gene for (G/A)(G/A)-N_19_-GG, and identify targets with >50% G/C content downstream of the empirically- or algorithmically-determined start codon.

To aid in target site selection, we developed an online webtool named CHOPCHOP (<https://chopchop.rc.fas.harvard.edu/>), which allows easy visualization of candidate Cas9 target sites within the gene, and predicts specificity using an algorithm that searches the genome for off-target sites.

As an example, here is the result of a query in CHOPCHOP for the *ctgfa* gene in zebrafish. We will follow this gene through the mutagenesis pipeline in this protocol.

First, access the website listed above and type ‘ctgfa’ into the search box. Next, click ‘Toggle advanced options,’ select CRISPR, and click the radio button requiring a 5’ GG. Now click “Find Target Sites,” which produces a window of results. The screen shot below indicates the position of the target sites on the *ctgfa* gene.


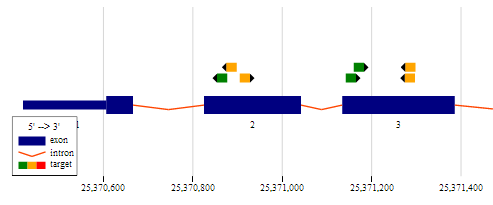


Selected target site

The highest scored target site for this sgRNA is GGGGCGGCACTTCAGGGCAGTGG. It was selected because of high G/C content, a guanine base adjacent to the protospacer adjacent motif (PAM), and because it is at the 5’ end of the open reading frame.

The first twenty bases (GGGGCGGCACTTCAGGGCAG) are the spacer region, and will be included in the sgRNA. The PAM is the last three bases (TGG). The PAM is not included in the sgRNA sequence, but is required in the genome for Cas9 targeting.

**Generate sgRNA**


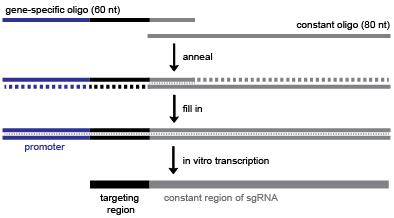


Above is a diagram (also Supplemental Figure S1) that describes our protocol for cloning-independent generation of sgRNAs. For each sgRNA, the user must order a 60 base oligonucleotide (“gene-specific oligo”) containing 1) a promoter for in vitro transcription (blue), 2) the 20 base spacer region specific to the target site (black), and 3) an overlap region that anneals to the constant oligonucleotide (grey). The user must order one gene-specific oligo per target and the 80 base constant oligonucleotide (the same for all sgRNA templates).

The user can select the T7 or SP6 (**recommended**) promoter.

T7: TAATACGACTCACTATA

SP6: ATTTAGGTGACACTATA

Overlap region: GTTTTAGAGCTAGAAATAGCAAG

The gene-specific oligo using SP6 will have the architecture shown below, with the Ns replaced with the 20 bases specific to the target:

ATTTAGGTGACACTATA-**N_20_-**GTTTTAGAGCTAGAAATAGCAAG

The gene-specific oligo for the example gene *ctgfa* has this sequence:

ATTTAGGTGACACTATAGGGGCGGCACTTCAGGGCAGGTTTTAGAGCTAGAAATAGCAAG

The constant oligonucleotide, regardless of promoter or target gene choice has this sequence:

AAAAGCACCGACTCGGTGCCACTTTTTCAAGTTGATAACGGACTAGCCTTATTTTAACTTGCTATTTCTAGCTCTAAAAC

We order standard desalted oligos from Life Technologies or IDT, with no additional purification.

*Anneal oligos:*

Gene-specific oligo (100 µM) 1 µl

Constant oligonucleotide (100 µM) 1 µl

Water 8 µl

Total: 10 µl

95˚C 5 minutes

95˚C -> 85˚C -2˚C/second

85˚C -> 25˚C -0.1˚C/second

4˚C hold

*Add T4 DNA polymerase to fill-in:*

dNTPs (10 mM) 2.5 µl

10x NEB buffer 2 2 µl

100x NEB BSA 0.2 µl

T4 NEB DNA polymerase 0.5 µl

Water 4.8 µl

Total 10 µl

Incubate for 20 minutes at 12˚C

Purify the template using a PCR cleanup column, and elute in 30 µl of water (expected: 100-200 ng/µl DNA)

Run on an agarose gel to verify a product of the correct size (dominant band should be ~120 bp).

*Transcribe sgRNA* (we use Ambion MEGAscript T7/SP6 Kit):

ATP 0.5 µl

GTP 0.5 µl

CTP 0.5 µl

UTP 0.5 µl

10x buffer 0.5 µl

T7/SP6 enzyme mix 0.5 µl

Water 1 µl

Template 1 µl

Total: 5 µl

Assemble in 1.5 ml tubes (or use 96-well plate). Incubate for 2-4 hours at 37˚C.

Add 14 µl water.

Add 1 µl TURBO DNase (included in Ambion transcription kit), incubate for 15 minutes at 37˚C.

*Cleanup sgRNA transcription reaction*

For high-throughput purposes, use a 96-well plate RNA cleanup kit. Otherwise use standard ethanol / ammonium acetate protocol, detailed below.

Add 10 µl 5 M ammonium acetate (included in transcription kit) and 60 µl 100% ethanol.

Mix well, incubate for 20 minutes at -80˚C until frozen.

Centrifuge for 15 minutes at 4˚C, maximum speed.

Remove the supernatant and add 1 ml 70% ethanol.

Centrifuge for 5 minutes at 4˚C, maximum speed.

Remove supernatant, dry at room temperature for a few minutes and resuspend in 50 µl water.

Determine RNA concentration (we use Nanodrop).

**Generate Cas9 mRNA or Protein**

Both Cas9 mRNA and protein can induce high indel frequencies; however, in our experience direct injection of the Cas9 protein / sgRNA complex leads to higher rates of mutagenesis. Be aware that preparing this protein yourself requires some expertise in protein expression and purification. Alternatively, Cas9 protein is commercially available from some vendors, although we have not tested its activity yet.

**Generate Cas9 mRNA**

We suggest using the pCS2-Cas9 plasmid to generate Cas9 mRNA -<http://www.addgene.org/Alex_Schier/>. This plasmid includes the SV40 polyadenylation signal.

Transform and grow the plasmid in liquid LB/ampicillin under standard conditions, and prepare DNA using a standard miniprep protocol.

*Linearize the plasmid*

Plasmid DNA 31 µl

10x BSA 4 µl

10x NEB Buffer 3 4 µl

NotI enzyme 1 µl

Mix and incubate for 1 hour at 37˚C, then purify using standard DNA column purification protocol.

Verify linearization and yield by running a sample on a 1% agarose gel with a DNA ladder for reference. The linearized pCS2-Cas9 plasmid is 8.2 kbp in size.

*Transcribe capped Cas9 mRNA using SP6 polymerase* (we use Ambion mMessage mMachine).

Linearized DNA plasmid 6 µl

2x NTP/cap mix 10 µl

10x transcription buffer 2 µl

10x SP6 enzyme mix 2 µl

Mix and incubate at 37˚C for 4 hours, then add 1 µl TURBO DNase (included in transcription kit) and incubate for 15 minutes at 37˚C.

*Cleanup transcription reaction* using standard ethanol / ammonium acetate precipitation

Add 10 µl 5 M ammonium acetate (included in transcription kit) and 60 µl 100% ethanol.

Mix well, incubate for 20 minutes at -80˚C until frozen.

Centrifuge for 15 minutes at 4C, maximum speed.

Remove the supernatant and add 1 ml 70% ethanol.

Centrifuge for 5 minutes at 4˚C, maximum speed.

Remove supernatant, dry at room temperature for a few minutes and resuspend in 50 µl water.

Quantify RNA concentration (we use Nanodrop), and run a sample on an agarose gel with a DNA ladder (full length Cas9 mRNA runs roughly equivalent to 1.5-2 kbp DNA).

Aliquot Cas9 mRNA at 600 ng/µl in 2 µl volume, store aliquots at -80˚C.

**Generate Cas9 protein**

For *in vitro* expression of Cas9 protein, the Cas9 open reading frame was cloned into pET-28b to generate pET-28b-Cas9-His. This plasmid is available from Addgene (<http://www.addgene.org/Alex_Schier/>) and allows simple expression and purification of Cas9 using His-tag / nickel column purification.

Transformed the plasmid into *E. coli* Rosetta cells (Novagen) using standard techniques and plate on LB/kanamycin/chloramphenicol. Cas9-His is expressed using the auto-induction method below, adapted from Studier F.W., *Protein Expression and Purification* 2005.

Pick a fresh colony into 3 x 1 liter ZYP-5052 + kanamycin + chloramphenicol cultures (see ZYP-5052 instructions below) and grow for 12 hours at 37˚C, followed by 24 hours at 18˚C, shaking at 200 rpm. Pellet cells using centrifugation (spin each 1 liter culture incrementally), and resuspend each cell pellet in 20 ml of wash buffer (recipe below).

Lyse cells using a sonicator with a cell disruptor tip with 120 seconds of sonication in 20-second intervals with 20 seconds rest between pulses. Mix cells briefly and repeat the lysis process to ensure complete lysis. Spin again at >14,000 rpm to pellet the cellular debris.

Prepare a column with 1-2 ml of nickel resin (G Biosciences). Load 1-2 ml resin onto the column, allow storage buffer to flow through by gravity, and rinse the resin twice with 10 ml of wash buffer.

Add the supernatant to the column and incubate on a rotating platform for 15-30 minutes. Allow the supernatant to flow through by gravity, keeping a sample of the flow-through for later analysis. Wash the column with 80 ml of wash buffer (recipe below), in 20 ml increments.

Elute with 1 ml increments of the elution buffer (recipe below). We do six elutions. Run a protein gel and stain with Commassie solution or equivalent to identify elution fractions with the highest concentration of Cas9. In our experience, the vast majority comes off in fractions 2 and 3. Dialyze the selected fractions into dialysis buffer using dialysis cassettes.

Quantify with a Bradford assay or A280 on Nanodrop. Single-use 2 µl aliquots should be frozen in liquid nitrogen and stored at -80˚C.

ZYP-5052 media can be purchased from Amresco, or made in the lab by following the instructions of Studier 2005 – see <http://www.ncbi.nlm.nih.gov/pubmed/15915565>

Detailed recipes provided here - <http://www.bnl.gov/biosciences/staff/Studier/files/StudierRecipes07-12-20.pdf>

Wash buffer : 20 mM Tris pH 8, 30 mM Imidazole, 500 mM NaCl

Elution buffer: 20 mM Tris pH 8, 500 mM Imidazole, 500 mM NaCl

Dialysis buffer: 20 mM Tris, 200 mM KCl, 10 mM MgCl_2_

**Design stop codon cassette oligonucleotide**

The stop codon cassette oligonucleotide contains two 20 base homology arms that flank the predicted Cas9-mediated breakpoint. These homology arms surround the stop codon cassette. The stop codon cassette v1 (used in Gagnon et al., 2014) has the sequence 5’-GTCATGGCTAATTAATTAAGCTGTTGTAG-3’. This cassette has stop codons in all frames, and also contains a PacI restriction enzyme site, as diagrammed in Figure 4 of Gagnon et al., 2014.

Note that we have recently updated to stop codon cassette v2, which has the longer sequence 5’- GTCATGGCGTTTAAACCTTAATTAAGCTGTTGTAG-3’. This cassette has stop codons in all frames, and also PmeI and PacI restriction enzyme sites. The stop codon cassette v2 also has the advantage of being a better template for an insert-specific PCR primer.


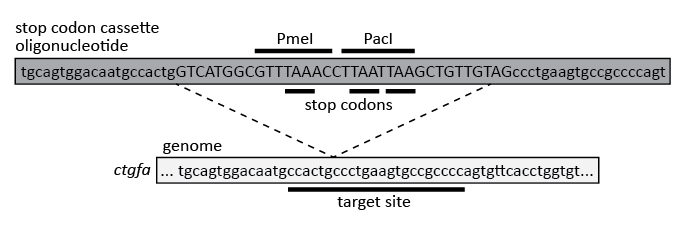


This is the design for stop codon cassette oligonucleotide v2 for the *ctgfa* gene:

Left homology arm sequence: tgcagtggacaatgccactg

Right homology arm sequence: ccctgaagtgccgccccagt

Combining these homology arms with the stop codon cassette v2 gives the following oligonucleotide:

tgcagtggacaatgccactgGTCATGGCGTTTAAACCTTAATTAAGCTGTTGTAGccctgaagtgccgccccagt

We order standard desalted oligos from Life Technologies, with no additional purification. Resuspend in 10 mM Tris pH 8 at 100 µM.

**Inject Cas9, sgRNA, and stop codon cassette oligonucleotide**

On the day of injection, first prepare the injection mix:

sgRNA (200-400 ng/µl) 1 µl

Cas9 protein or Cas9 mRNA (600 ng/µl) 1 µl

3 µM stop codon cassette oligonucleotide 1 µl

phenol red injection dye 0.3 µl

For Cas9 protein, mix components and incubate at room temperature for 5 minutes to form the complex, then store on ice. For Cas9 mRNA, mix and store immediately on ice.

Load the injection mix into the needle and microinject into zygotes using standard zebrafish injection protocols. We aim to inject 1 nL into the cell (not the yolk), and take care to make sure each embryo is correctly injected. We inject through the chorion for five reasons-

1. Embryos can be injected immediately after collection – no need to dechorionate,
2. Embryos are easier to move from dish to dish and grow at higher density when still in the chorion,
3. Embryos have a better survival rate in the chorion, especially after perturbation,
4. Embryos inside the chorion are better protected during growth from neighboring dead embryos than dechorionated embryos, and
5. It is easier to score survival when dead embryos are contained in the chorion.

Keep some uninjected embryos from the same clutch, and grow the embryos at 28.5˚C. **Determine somatic indel frequency**

At 24-30 hpf, remove all “monsters” and dead embryos. If the injection was too toxic (<50% normal embryos), try again with lower concentrations of Cas9 protein / sgRNA /oligonucleotide.

*Prepare genomic DNA* using the HotSHOT protocol (Meeker, Hutchinson et al., *Biotechniques* 2007)

Select 10 of the remaining injected embryos and 10 uninjected embryos. There is no need to dechorionate the embryos before preparing genomic DNA.

- 1. Move embryos in blue water to a labeled PCR strip, plate or tubes
  2. Remove blue water
  3. Add 100 µl 50 mM NaOH
  4. Incubate for 20 minutes at 95˚C
  5. Cool to 4˚C
  6. Add 10 µl 1 M Tris HCl pH 7.5
  7. Mix

*Two-step fusion PCR to prepare for sequencing by MiSeq –* Determining indel frequency by deep sequencing is most appropriate for screening many sgRNAs at the same time. Users interested in only a small number of sgRNAs can use a variety of other published methods of determining indel frequency, such as T7 Endonuclease I assay, HRMA, restriction enzyme digest, PCR with insert-specific PCR, etc.


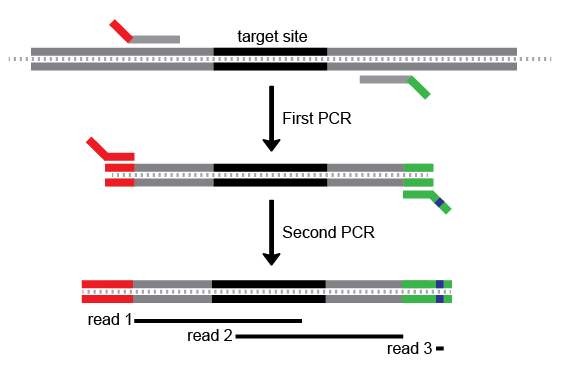


A diagram of the two-step fusion PCR for library preparation. Target site in black. The first PCR step uses gene specific primers (grey) with extensions for the second PCR step, which uses universal primers to attach the sequence adapters (red and green, with barcode in blue). In this example only one barcode is shown, but many can be used – we’ve used up to 24. Finally, three sequencing reads cover the amplicon and the barcode.

First PCR with target gene-specific primers:

This amplifies the genomic region surrounding any mutations. Keep the size of the first amplicon small – less than 280 base pairs - so that 150 base sequencing reads from both ends can overlap the predicted Cas9 cleavage site. These primers also have extensions for priming in the second PCR (see diagram, and example below). We design our primers for a 66C annealing temperature.

| 5xHF buffer | 2 µl |
| --- | --- |
| 10 mM dNTPs | 0.2 µl |
| water | 5.7 µl |
| 5 µM F/R primer mix | 1 µl |
| Phusion | 0.1 µl |
| Genomic DNA | 1 µl |
| **Total** | **10 µl** |

| 98˚C | 30 sec |
| --- | --- |
| 98˚C | 10 sec  30 cycles |
| 66˚C | 20 sec |
| 72˚C | 20 sec |
| 72˚C | 3 minutes |
| Hold at 4˚C |  |

Verify that you have a band of the correct size on an agarose gel before proceeding to the second PCR.

For *ctgfa­,* the two primers for the first PCR are listed below, with the universal extensions in lower case and the gene-specific segment in upper case:

ctgfa F: tctttccctacacgacgctcttccgatctTGATCAAGCTAGGCTTAAAATGGAG

ctgfa R: tggagttcagacgtgtgctcttccgatctGGTCGCAAACATCTCGTTCTG

This generates a 260 base pair amplicon for *ctgfa*.

Second PCR with barcoding primers:

This PCR uses universal primers to attach the sequencing adapters and barcode to your amplicon (see diagram above). The forward primer is always the same (P5 F), but we have two different reverse primers containing different barcodes. This barcoding allows comparison between injected and uninjected indel frequencies for the same amplicon. Set up a PCR using injected amplicon with Barcode 6 R primer, and using uninjected amplicon with Barcode 12 R primer.

| 5xHF buffer | 2 µl |
| --- | --- |
| 10 mM dNTPs | 0.2 µl |
| water | 5.7 µl |
| 5 µM F / R primer mix | 1 µl |
| Phusion | 0.1 µl |
| 1:4 diluted first PCR | 1 µl |
| **Total** | **10 µl** |

| 98˚C | 30 sec |
| --- | --- |
| 98˚C | 10 sec  20 cycles |
| 66˚C | 20 sec |
| 72˚C | 20 sec |
| 72˚C | 3 minutes |
| Hold at 4˚C |  |

Again, verify a band of the correct size on a gel – this amplicon will be 65 base pairs larger than the previous amplicon because of the added sequencing adapters. We sometimes see a secondary product around ~175bp in addition to the correct band; this is the result of amplification of primer-dimers from the 1^st^ round of PCR.

Barcoded primers are from Illumina TruSeq library suggestions, and are given below:

P5 F: AATGATACGGCGACCACCGAGATCTACACTCTTTCCCTACACGACGCT

Barcode 6 R : CAAGCAGAAGACGGCATACGAGAT**TATTGGC**GTGACTGGAGTTCAGACGTGTGCT

Barcode 12 R : CAAGCAGAAGACGGCATACGAGAT**TTACAAG**GTGACTGGAGTTCAGACGTGTGCT

Barcodes are in bold. More barcodes are available, check the Illumina TruSeq guidelines for details.

This generates a 325 bp amplicon for *ctgfa*.

Pool and gel extract amplicons

Pool amplicons from different clutches – but do not pool across amplicons of different sizes as this makes gel extraction in the next step more difficult. From the previous gel, try to estimate equal molarities when pooling (i.e. compensate for weak amplicons by adding more to the pool). For a strong amplicon, even 1 µl of PCR product can be enough.

Pour a 2% agarose gel and gel extract all the pooled amplicons using a gel extraction kit.

Make sequencing library

Determine the concentrations of amplicon pools. Combine equal molarities of each pool to form a 10 nM sequencing library. We usually spike-in 5% phiX DNA as suggested by Illumina. We sequence this library on MiSeq using MiSeq Reagent Nano Kit v2 (Illumina) according to the manufacturer’s instructions.

Determine the indel frequencies as described in the Methods section of Gagnon et al., 2014.

**Outcross adult injected fish, determine germline indel frequency and allele identity**

Once injected fish are grown to fertile adults, outcross putative founders and screen clutches for the inserted stop codon cassette by PCR as described below.

Prepare genomic DNA from 5-20 embryos at 24-30 hpf as described above.

Screen this genomic DNA using PCR with one gene-specific primer and a primer specific to the inserted sequence.

| 5xHF buffer | 2 µl |
| --- | --- |
| 10 mM dNTPs | 0.2 µl |
| water | 5.7 µl |
| 5 µM F / R primer mix | 1 µl |
| Phusion | 0.1 µl |
| Genomic DNA | 1 µl |
| **Total** | **10 µl** |

| 98˚C | 30 sec |
| --- | --- |
| 98˚C | 10 sec  35 cycles |
| 66˚C | 20 sec |
| 72˚C | 20 sec |
| 72˚C | 3 minutes |
| Hold at 4˚C |  |

Run on a 2% agarose gel looking for a band of correct size.

*Ctgfa* gene-specific F: TGATCAAGCTAGGCTTAAAATGGAG

Universal insertion-specific R: CAACAGCTTAATTAAGGTTTAAACGCCATG

For *ctgfa*, this amplicon is approximately 144 bp.

Once a clutch has been confirmed to have germline transmission of the inserted stop codon cassette, the remaining embryos can be grown to adults to identify heterozygous adults.

**Identify adult heterozygous individuals and allele identity**

Anaesthetize and fin clip putative heterozygotes. Use PCR as described above to determine the heterozygous individuals. Because of the nature of the stop codon cassette, any insertion should cause truncation of the targeted open reading frame. However, we recommend cloning and Sanger sequencing the allele for confirmation.
